# Barriers and facilitators in implementing a pilot, pragmatic, telemedicine-delivered healthy lifestyle program for obesity management in a rural, academic obesity clinic

**DOI:** 10.1186/s43058-020-00075-9

**Published:** 2020-09-30

**Authors:** John A. Batsis, Auden C. McClure, Aaron B. Weintraub, Diane Sette, Sivan Rotenberg, Courtney J. Stevens, Diane Gilbert-Diamond, David F. Kotz, Stephen J. Bartels, Summer B. Cook, Richard I. Rothstein

**Affiliations:** 1 Division of Geriatric Medicine, Department of Nutrition, University of North Carolina at Chapel Hill, Chapel Hill, NC USA; 2 Geisel School of Medicine at Dartmouth and The Dartmouth Institute for Health Policy & Clinical Practice, Lebanon, NH USA; 3 Dartmouth Weight & Wellness Center, Lebanon, NH USA; 4 Department of Psychiatry, Dartmouth-Hitchcock, Lebanon, NH USA; 5 Dartmouth Centers for Health and Aging, Dartmouth College, Hanover, NH USA; 6 Department of Epidemiology, Dartmouth College, Hanover, NH USA; 7 Department of Computer Science, Dartmouth College, Hanover, NH USA; 8 Mongan Institute, Massachusetts General Hospital, Boston, MA USA; 9 University of New Hampshire, Durham, NH USA

**Keywords:** Obesity, Telemedicine, Rural, Weight loss, Pragmatic

## Abstract

**Purpose:**

Few evidence-based strategies are specifically tailored for disparity populations such as rural adults. Two-way video-conferencing using telemedicine can potentially surmount geographic barriers that impede participation in high-intensity treatment programs offering frequent visits to clinic facilities. We aimed to understand barriers and facilitators of implementing a telemedicine-delivered tertiary-care, rural academic weight-loss program for the management of obesity.

**Methods:**

A single-arm study of a 16-week, weight-loss pilot evaluated barriers and facilitators to program participation and exploratory measures of program adoption and staff confidence in implementation and intervention delivery. A program was delivered using video-conferencing within an existing clinical infrastructure. Elements of Consolidated Framework for Implementation Research (CFIR) provided a basis for assessing intervention characteristics, inner and outer settings, and individual characteristics using surveys and semi-structured interviews. We evaluated elements of the RE-AIM model (reach, adoption) to assess staff barriers to success for future scalability.

**Findings:**

There were 27 patients and 8 staff completing measures. Using CFIR, the intervention was valuable from a patient participant standpoint; staff equally had positive feelings about using telemedicine as useful for patient care. The RE-AIM framework demonstrated limited reach but willingness to adopt was above average. A significant barrier limiting sustainability was physical space for intervention delivery and privacy and dedicated resources for staff. Scheduling stressors were also a challenge in its implementation.

**Conclusions:**

The need to engage staff, enhance organizational culture, and increase reach are major factors for rural health obesity clinics to enhance sustainability of using telemedicine for the management of obesity.

**Trial registration:**

Clinicaltrials.gov NCT03309787. Registered on 16 October 2017.

Contributions to the literature

- Technology-delivered care can be helpful in scaling interventions for rural adults with obesity residing in remote areas
- Policymakers can enhance reimbursement and incentives to promote telemedicine-delivered care to adults
- Enhancing patient/staff engagement and culture can overcome organizational stress and enhance reach of the intervention

## Background

Tertiary care weight management clinics located at academic medical centers usually serve patients from large geographic areas. Travel becomes a significant service barrier to adults residing in remote and isolated areas, and hence, disorders such as obesity may not be perceived by patients as urgent or harmful [1]. Routinely integrating telemedicine into clinical infrastructures may provide an opportunity to address these hurdles. Telemedicine can increase the exposure for low-resource populations without access to specialized services, particularly in conditions that do not necessarily require physical touch.

Previous health behavior change interventions using telemedicine effectively improved readiness to change [2] and provided effective psychiatric treatment [3]. Recognizing the need for different models of health service delivery, we conducted a pragmatic clinical pilot trial within our weight-management clinic aimed at providing usual clinical care using two-way, live, telemedicine. Our results were proven to be feasible, acceptable, and potentially effective at producing weight loss [4]; yet, this delivery system (telemedicine) could potentially lead to inherent challenges for both patient and provider stakeholders. Successful implementation of evidence-based practices requires an understanding of the barriers and facilitators of implementing new services. Two well-recognized frameworks for exploring implementation factors, barriers/enablers that permit planning, evaluating practice change interventions, and why implementation succeeded or not to identify modifiable factors, are the Consolidated Framework for Implementation Research (CFIR) and RE-AIM [5]. These frameworks have previously been applied in the context of telemedicine and have found that patient and leadership engagement, comfort levels with the technology, and a need to have similar efficacy and workflows to in-person visits were important factors [6–8]. As such, the purpose of this analysis was to apply these established implementation frameworks to refine our intervention for a future, large-scale trial that could ensure its long-term success and program sustainability in rural areas. We anticipate that our findings could provide useful guidance to others in implementing evidence-based practices.

## Methods

### Study setting

Dartmouth-Hitchcock (D-H) is a 396-bed hospital affiliated with Dartmouth College, an Ivy League University, and is New Hampshire’s only teaching hospital. Ambulatory services provide care for 1.5 million patients yearly, consisting of 1.3 million outpatient visits. This rural medical center is located in Lebanon, New Hampshire, USA, a small town of 13,522 persons, within Grafton County (population 89,396) [9], adjacent to the Vermont border in the northeastern USA. The Center for Telehealth leveraged institutional resources to assist with setup, deployment, and use of telemedicine throughout the study and was responsible for orientation, training, and setup of the software; hardware requirements; configuration of the tablets; and support, with troubleshooting back-up provided by the research assistant for any difficulties.

### Intervention study design and description

The clinical trial was conducted between December 2017 and September 2018 within the Weight & Wellness Center clinic. During this time, the clinic saw 385 new consultations for adult obesity management. The study was approved by the Committee for the Protection of Human Subjects and the Dartmouth-Hitchcock Institutional Review Board and was registered at clinicaltrials.gov #NCT03309787. The trial was a single-arm, evidence-based weight-loss intervention [10] consisting of an individualized 1:1 exercise and nutrition counseling behavioral change program. Participants were referred by their primary care provider. We enrolled 37 participants, of which 27 completed the intervention. Participants were approached by the on-site clinician, and if interested, a research assistant obtained informed consent. Study recruitment methods have previously been described [4].

The 16-week Healthy Lifestyle Program was based on the Diabetes Prevention Program [11] focusing on health-behavior change delivered using individual, 1:1 sessions with trained staff (see below) using telemedicine. Patients underwent a comprehensive multidisciplinary intake. Visits lasted 30 min each, conducted by the interventionists in the clinic using video-conferencing. Each used motivational interviewing, goal-setting, and coaching strategies for the telemedicine participants to parallel the on-site program being delivered for clinical care. Supplemental File #1 presents the specific curriculum. We previously demonstrated that this program was acceptable and effective in delivering the in-person program using telemedicine on weight loss [4].

### Intervention implementation

#### Study design

The aim of this current analysis and manuscript was to provide information of the telemedicine-delivered intervention in the pre-implementation phase that could inform a future, large-scale intervention and could provide useful information to other rural obesity practices. For this component of the study, study participants and staff completed questionnaire measures (see below) and a semi-structured exit interview. The Standards for Reporting Implementation Studies are in supplemental file #2 [12].

#### Study participants

Study sessions were offered by a health coach, nurse exercise specialist, and registered dietitian each of whom were licensed and trained, having experience working with patients with obesity for at least 5 years. An administrative assistant and a clinical secretary were responsible for scheduling patient participants and was conducted as per usual clinical care guidelines and provider schedules. A practice manager oversaw the clinical operations and managed staff within the clinic. Two physicians and an advanced practice registered nurse informed prospective participants of the study during their initial, in-person consultations. A research assistant was responsible for all project management tasks, including troubleshooting of the encounters, data entry and management, and analysis. There were 27 study completers and 8 staff who completed post-intervention follow-up survey measures and an exit interview on the barriers and facilitators of the intervention. Interviews were all conducted by the lead author (JAB) and were structured using the above frameworks. Questions posed to staff are presented in Supplementary File #3 and those to patient participants in Supplementary File #4.

#### Implementation assessment frameworks

We explored barriers and facilitators, including elements from the Consolidated Framework for Implementation Research (CFIR) [13] and the RE-AIM (Reach, Effectiveness, Adoption, Implementation, Maintenance) [14] frameworks.

#### CFIR outcome measures

We evaluated the *intervention characteristics* using a satisfaction questionnaire (Table 1) to patient participants (1–5 Likert scale, low to high). To measure the *inner setting*, staff (*n* = 8) completed a 38-measure questionnaire [15] that evaluated culture, culture stress, culture effort, implementation climate, learning climate, leadership engagement, and available resources (Table 2, Supplemental File #4). We assessed organizational change using an adapted version (Supplemental File #5) of the General Organizational Index [16] administered to staff. This scale is an interview of 11 domains with a 5-point rating of program philosophy, commitment, client eligibility and identification, health promotion plan and its treatment, training, process and outcome monitoring, assessment, quality assurance, choice supervision, and penetration. The *Outer setting and Characteristics of the Individuals* involved single questions (range 1–10) evaluating whether staff had confidence in the intervention delivery (Table 3). Interviews explored the perceived advantages, disadvantages, and value of the pilot both to patient participants and staff, the latter previously published [4]. We qualitatively assessed staff barriers to success for future scalability (Table 4).

**Table 1:** Intervention characteristics—patient perspective of ease of use and value of technology (*n* = 27)

	Mean ± SD^a^	Median	Range
**Ease of use of technology**			
Did you feel that the overall intervention was beneficial and worth your time?	4.6 ± 0.7	5	2–5
How would you rate your level of satisfaction with the video-conferencing device?	4.4 ± 0.8	5	2–5
How helpful was video-conferencing in assisting you to achieve your goals?	4.4 ± 0.7	4	2–5
Did you find the video-conferencing easy to use without much difficulty?	4.9 ± 0.3	5	4–5

*SD* standard deviation

^a^Scales are represented on a Likert of 1–5, ranging from low to high

**Table 2:** Staff inner setting evaluation using Fernandez evaluation (*n* = 8) [15]

Subscale	# Questions	Mean	Median	Range	Maximum survey score
**Organizational culture**	9 questions	39.9 ± 3.2	40	36–44	45
**Culture stress**	4 questions	12.3 ± 2.7	12.5	8–16	20
**Culture effort**	5 questions	23.5 ± 1.5	23.5	21–25	25
**Implementation climate**	4 questions	14.1 ± 2.0	14	11–18	20
**Learning climate**	5 questions	21.3 ± 3.3	20	17–25	25
**Leadership engagement**	4 questions	13.5 ± 2.1	14	10–16	20
**Available resources**	7 questions	22.1 ± 2.2	22	20–27	35
**Total**	**38 questions**	**146.6 ± 8.6**	**143.5**	**139–163**	**190**

Mean ± standard deviation, median, and range are listed for the measures. The last column represents the maximum score for each subscale. This is a 38 item measure evaluating culture, culture stress, culture effort, implementation climate, learning climate, leadership engagement, and available resources. Supplemental File #3 outlines each subscale’s detailed questions and scoring from each component

**Table 3:** Staff confidence in the intervention delivery (*n* = 8)

	Mean ± SD	Median	Range
My “buy-in” was very high for this project	6.9 ± 3.0	8.0	3–10
I promoted the Telehealth Project to Patients	7.3 ± 2.8	8.0	2–10
I think that Telehealth can improve care quality	7.6 ± 3.1	8.5	1–10
I think the clinic is ready to adopt telemedicine in one form or another	7.3 ± 3.1	8.5	3–10
I value telemedicine as an emerging technology	9.1 ± 1.1	9.5	7–10

Scores range from 1–10 to low to high

**Table 4:** Linking CFIR/RE-AIM to thematic analysis of staff and patient participant perceptions of intervention

CFIR	Domain	Staff Representative Quote	Patient Participant Representative Quote
**Intervention characteristics**	**Advantages**	It allows us to provide our intensive lifestyle therapy to those unable to be here on a weekly basis.	It definitely saves you in traveling and trying to schedule an appointment to come down, and so you don’t have to get out of work to take an appointment or whatever, so it’s definitely easier scheduling wise.
**Inner setting**	**Workflow interference**	Space was a big one, and tight scheduling was another one, and sometimes that was back-to-back telemedicine visits where patients were scheduling us in around their workday, and so they didn’t necessarily have the flexibility to start late or whatnot	The dietitian was way behind on schedule and caused me to miss [a session] one day
	**Change in work hours**	There were occasions when I [administrative staff] didn’t get lunch.	----
	**Barriers to success**	We need a clinic place where we can actually think and not be interrupted and be able to be setup appropriately for this. That was a huge struggle for me in particular because I don’t have a room.	Sometimes I found that like when, like the health coach or dietitian, they were going through their spiel and I wanted to put my little two sense in, they were on their roll. And it was hard to get that in. Where maybe if you were face to face, they’d read that body language that you had something you wanted to say. That would be my only negative to that.
		It was just the exhaustion that I’ve repeated myself for 30 minutes of information	I think, it really depends on kind of the provider having, being ready and having their material ready. I think people were pretty good in this study but, yeah, it’s a little, it’s maybe a little stiffer. It’s a little harder to kind of have a back and forth sometimes, but.
		There should be more flexibility in the scheduling; especially not having back-to-back times, even if there was a 15-minute buffer in there following an appointment to allow for (i.e., tight scheduling)	The disadvantages is, that I felt like I had a make my schedule all around this, and they, we did switch to, and they switched too
	**Enablers and facilitators**	I think it’s a great way to deliver this information and a great way [convenience] to access those people who can’t make it in here	They give you exercises, they give you nutrition information, they give you health coaching information, like ways to help deal with stressful eating and fast hurried eating, not paying attention eating. Then you go and test when you start, and then you get to test again when you end and see how much you’ve progressed.
		Telemedicine saves on transportation, time, mobility.	As long as they’ve got connections, it can save them the drive
		I think it’s a way to do a quick check-in, if you will, an assessment. It’s kind of another touchpoint where they can feel connected.	Definitely it can help you keep in touch with the provider instead of having to make an appointment, you can get ahold of them.
		For a relationship perspective, because you don’t have the in-person face-to-face thing, you do lose energy, and only see part of the person.	Well, the lack of human support, the supportive group concept, the weighing in piece
**Outer setting**	**External policies**	From a billing standpoint, we potentially lost four dietitian billable appointments per person,	–
**Characteristics of individuals**	**Motivation**	It really depends largely on the patient participants’ readiness for change to participate	It’s a stepping stone to kick-start some motivation, and to definitely increase stream of consciousness. If somebody is unsure how to begin a program, this is certainly a good framework for helping them get started.
**RE-AIM**			
**Reach**	**Effect on number of patients treated**	I’m not sure how it would differ in terms of versus a class, where you could fit 14 to 15 people in an hour. I still think the group model either way is a great one and less resource intense.	I don’t know, maybe like a group session [is needed], maybe each quarter throughout the program
**Adoption**	**Workflow interference**	We would have to take a closer look at the workflow related to scheduling and the space before I would I say, “Yeah, I’d love to do telemedicine.”	–

#### RE-AIM assessment measures

Using RE-AIM, we estimated *Reach*, which consisted of the proportion of the number enrolled to the number evaluated for consultation during this time. Haug’s staff *Adoption* questionnaire (Table 5) [68] permitted quantitative evaluation of the stage of change, experience, attitudes, organizational barriers, and strategies to support evidence-based practices (Likert 1–5). Perceived staff workflow delays were also evaluated (Likert 1–10 scale). Qualitative interviews inquired about the patient and staff’s experience with the intervention; for staff, we explored whether it enhanced or interfered with workflow, could be sustainable, or if there were other technical or other difficulties in delivering care.

**Table 5:** Staff adoption questionnaire [17] (*n* = 8)

	Mean ± SD	Range
**Staff adoption questionnaire**		
**Attitudes—positive outcome**		
Using a treatment manual helps a therapist to evaluate and improve his or her clinical skills^a^	3.6 ± 0.5	3–4
Following a treatment manual will enhance therapeutic outcomes by insuring that the treatment being used is supported by research^a^	3.4 ± 0.7	2–4
If a treatment has been shown scientifically to be effective, then the counselor is ethically obligated to use the treatment as opposed to one that has not been studied^a^	3.3 ± 1.0	2–5
**Attitudes—negative process**		
Evidence-based practices make counselors more like technicians than caring human beings	3.6 ± 1.2	2–5
Treatment manuals are appropriate for research clients but not “real world” clients	3.5 ± 0.9	2–5
Using evidence-based practices detracts from the authenticity of the therapist interaction	4.0 ± 0.5	3–5
**Organizational barriers**		
Evidence-based practices seem overly complicated and hard to put into practice	3.8 ± 0.9	2–5
There are influential clinicians at my program that are definitely against evidence-based treatments.	4.4 ± 0.9	3–5
It would take some very strong incentives, such as restricting our funding, before our treatment program would use evidence-based practices	4.3 ± 0.9	3–5
The idea of evidence-based practices sound good in “theory,” but in reality, it is virtually impossible to scientifically test a phenomenon as complex as substance abuse treatment	3.9 ± 1.2	1–5
The treatments that we do at our program may not be “evidence-based,” but they work just as well, or better.	3.4 ± 1.2	2–5
As long as they do not conflict with treatments already in place at our program, I do not see any problem with using a few procedures that are evidence-based^a^	4.0 ± 0.5	3–5

1 strongly disagree; 5 strongly agree

*SD* standard deviation

^a^Reverse score

### Data analysis

Descriptive statistics were computed for continuous variables; for single item questions, proportions (where appropriate) were calculated. All interviews were digitally recorded in duplicate and transcribed. Data were aggregated into *Dedoose* [Hermosa Beach, CA]. Transcripts were read (JAB) who conducted open coding, with themes independently verified by ABW, an approach that enhances research rigor by allowing for different viewpoints [18]. A codebook was developed using researcher-driven codes derived from each interview and codes generated through an inductive review of the transcripts. Focused coding using themes identified during open coding permitted defined analyses through data immersion. A query tool retrieved the text by code, reviewed for content, relevance, and prevalence of themes and grouped. Themes and comments were mapped to specifically to barriers/facilitators of using telemedicine, including its impact on clinical care using pre-specified questions related to the specific elements of the framework. The main themes reported in this study were identified, reported, and aligned, where possible to CFIR/RE-AIM.

## Results

### Patient cohort

Of the 27 who completed > 75% of all sessions, mean participant age was 46.1 ± 12.3 years (88.9% female) with a body mass index of 41.3 ± 7.1 kg/m^2^. Mean weight loss was 2.22 ± 3.18 kg (2.1% change; *p* < 0.001). There was a loss in waist circumference of 3.4% (*p* < 0.001) [4]. The mean distance from the participant’s home to the center was 38.8 ± 31.6 miles (mean driving time 36.0 ± 29.0 min). Table 1 describes the intervention characteristics from the patient perspective, including the benefit of telemedicine (4.9/5), its value and ease of use (4.6/5), and its satisfaction and helpfulness (4.4/5). All were strongly favorable to patients. Patient participants had similar favorable sentiments regarding telemedicine as did staff (see below).

### Implementation measures

Data on the Inner Setting Evaluation is presented in Table 2 (Supplemental File #5). Both culture stress and available resources were challenging, while organization culture and effort to implement was strongly favorable. The adapted General Organizational Index [16] demonstrated two concerns: client identification and process monitoring. Staff confidence in intervention delivery is presented in Table 3. Generally, staff had positive feelings regarding using telemedicine and noted above average expectations regarding its usefulness (Table 4). Haug’s staff adoption questionnaire [17] is presented in Table 5. Results suggest an average to above-average willingness for staff to adopt telemedicine. While the range of answers were broad, the median response score was high, suggesting that staff felt confident that this intervention had considerable potential.

Table 5 represents the major domains and themes that emerged using select elements from the CFIR and RE-AIM frameworks. Representative quotes are presented including the usefulness of telemedicine, its advantages, and the loss of value that may occur using this technology. Space for conducting video-conferencing was a major barrier to success. Many believed that a hybrid intervention (part in-person, part remote) might reduce depersonalization. Dedicated resources from senior administration were needed to ensure successful delivery of telemedicine within the clinical framework.

Using RE-AIM, we evaluated reach and adoption. We estimated that this program only reached 37 participants with telemedicine (e.g., enrolled participants) of a potential 385 participants (9.6%) that needed treatment for obesity. Some crucial findings on workflow interference, the impact on reach, work hours, and barriers to success are also presented. Staff felt that the study did lead to workflow delays (mean 5.0 ± 1.7, median 5.0, range 2–7), but few believed their existing tasks were altered considerably (3.5 ± 3.4, median 1.5, range 1–9).

## Discussion

The importance of engaging patient and provider stakeholders in effectively delivering telemedicine within a clinical setting cannot be overstated. Our results highlight the characteristics needed for clinical execution prior to full-scale deployment in order to maximize scalability and dissemination. There was clear value to patient participants with minimal challenges of using telemedicine.

Staff faced several workflow delays as evidenced by our mixed-methods assessment. Workspace issues impacted all domains. While space is limited in many healthcare centers [19], telemedicine requires a dedicated infrastructure that includes a workspace free from distractions to effectively engage in the two-way conversation and preserve privacy. In our environment, health coaches are situated in a large workroom and hence video-delivery may lead to additional distractions beyond privacy concerns. The findings observed in this study parallel those observed by Brown who found that primary-care providers voiced concerns that staffing, space, and aligning the sessions on days/times when staff/patients were available [20] were barriers to telemedicine implementation. Our data suggest that staff engagement was highly reliant on the ability to adequately conduct and schedule such encounters. Whether specific computer peripherals can be used (i.e., noise-canceling headphones) is a possibility. The inability to integrate sessions within routine in-person care was a major impediment to success and highly dependent on several external factors. Providers are constrained in their ability to run on time based on rooming, scheduling, and patient arrival times that could lead to downstream delays. Increasing visit times or providing breaks between telemedicine sessions and in-person sessions may be a potential solution.

“Inner setting” factors impacting staff satisfaction included participants characteristics. All screened patients were offered the intervention. Readiness to change is a known determinant of attrition and intervention compliance for obesity treatment programs [21]. Our program did not screen participants on the basis of this measure, leading to participants who may have been less motivated to initiate change being included in the sample. Staff felt that 1:1 visits were not cost-effective and that group-based therapy would enhance provider satisfaction. Operationally group visits could improve downstream revenues. Such a hybrid in-person/remote program also adds the benefit of in-person touches and social connectedness that may not be observed with full-remotely delivered care. Generally, staff had confidence in this mode of delivery, although adopting this intervention without future changes would be problematic. These factors have previously been observed with the participants completing this intervention [4].

The evidence-based Veterans Affairs MOVE program [22, 23] and their telemedicine results have demonstrated favorable effectiveness outcomes [24]. Our preliminary results suggested similar challenges including the need for more staff time. A randomized trial testing a weight-loss intervention delivered via telehealth for rural cardiac rehabilitation patients (mean age 63 ± 9.3 years) also demonstrated usefulness and feasibility, but studies are markedly underpowered. Additional research is needed to further this modality with such populations [25].

Implementing a rigorous evaluation could enhance, scale, and expedite dissemination of research-level programs as evidenced by the TeleMOVE program [26]. Sustainability is most important, both operationally and financially. Without such information, it would be difficult to modify programs to permit long-term solvency. Additionally, our intervention was implemented in a culture conducive to process change. There was also engagement of the academic missions from senior stakeholders. Our investigation also had a number of limitations. First, our pilot was non-randomized and limited to eight personnel providing information on clinic-related implementation outcomes. While we acknowledge that it would be hard to implement a large-scale project using data from this small number of staff, it does provide the team with important information to conduct a larger, type I hybrid, effectiveness-implementation trial. Second, our results acknowledge the contextual and organizational factors that could impact future implementation. Third, we did not formally evaluate the fidelity of the intervention or other measures of implementation that could potentially foster long-term sustainability. Last, while the intervention provided partial support to a research coordinator, none of the clinical staff were reimbursed, in line with a pragmatic intervention. Whether other centers are similarly supported is unknown.

### Implementation implications for other rural health practices

While exploratory, our implementation outcomes provide useful information to scale-up and spread innovative healthcare interventions [27]. By incorporating our suggestions to address shortcomings, we can promote sustainability, success and improved patient outcomes [28–30]. We recognize that not all elements of CFIR or RE-AIM were evaluated and that this may limit our ability to effectively integrate specific elements that can be helpful in long-term dissemination. However, our experience provides formative information for others to consider in implementing telemedicine in other venues, including primary and specialty care using the above-noted frameworks:

- Disseminating an evidence-based intervention: This exploratory data demonstrates that the intervention characteristics (CFIR) from a provider and patient participant standpoint were favorable suggesting that the Healthy Lifestyle Program can be scaled favorably to other centers. Importantly, our results suggested that staff had favorable attitudes and confidence in adopting the intervention (RE-AIM).
- Patient/staff engagement: The lack of a face-to-face encounters led to depersonalization, suggesting a need and value of periodic in-person visits for patient. Rather than the entire intervention be conducted remotely, a hybrid in-person/remotely delivered program could promote social engagement and connectedness among participants, but also among staff (RE-AIM). However, only a future trial could best test this delivery strategy.
- Enhancing organizational culture: Our experience noted that mixing telemedicine visits with on-site visits led to consider stress and inefficiencies amongst staff (CFIR). Block scheduling (e.g., all telemedicine visits occurring in sequence) could easily overcome these challenges.
- Overcoming organizational stress: Key barriers to successful implementation of telemedicine-based interventions include the need for a dedicated space for telemedicine to eliminate potential distractions. Our experience suggested that conducting telemedicine in large workspaces leads to distractions and considerable stress to study staff (CFIR).
- Increasing reach: Current 1:1 sessions reduce the potential reach of participants and should be reserved for those needing personalized therapy (RE-AIM). Group remotely delivered sessions can reduce staff burden and fatigue but increase the ability to reach more people with limited resources.

## Conclusions

Applying implementation evaluation frameworks such as CFIR and RE-AIM in our pragmatic pilot intervention of a telemedicine-delivered wellness intervention provided data in our pre-implementation phase that could inform future trial sustainability and spread not only locally, but to other centers and other specialties. Our approach is novel in that it incorporates elements of implementation science in a rural academic infrastructure that, to our knowledge, has not been fully explored that could be helpful to enhance obesity care within a specialty, rural care environment to provide data on how to maximize the impact of practice-based interventions.

## Supplementary information

**Additional file 1: **Supplemental File 1: Components of the Healthy Lifestyle Program at the Dartmouth Weight and Wellness Center. Supplemental File 2 Standards for Reporting Implementation Studies: the StaRI checklist for completion. Supplemental File 3 – Staff Questions. Supplementary File 4 – Patient Satisfaction Questions. Supplemental File 4: Inner Setting Measures from the CFIR Framework – Fernandez et al (1-low to 5-high, strongly disagree to strongly agree): (*n*=8). Supplemental File 5 – Adapted General Organizational Index.

## Data Availability

The datasets generated and/or analyzed during the current study are not publicly available due to data privacy policies at Dartmouth-Hitchcock but may be available from the corresponding author on reasonable request.

## Abbreviations

CFIR: Consolidated Framework for Implementation Research
D-H: Dartmouth-Hitchcock
RE-AIM: Reach, Effectiveness, Adoption, Implementation, Maintenance
