# Supplementary material for: Barriers and facilitators in implementing a pilot, pragmatic, telemedicine-delivered healthy lifestyle program for obesity management in a rural, academic obesity clinic
Source: Implement Sci Commun. 2020 Sep 30;1:83. doi: 10.1186/s43058-020-00075-9 (PMC7526351; doi:10.1186/s43058-020-00075-9)
Supplement: Supplementary file 1 — Additional file 1: Supplemental File 1: Components of the Healthy Lifestyle Program at the Dartmouth Weight and Wellness Center. Supplemental File 2 Standards for Reporting Implementation Studies: the StaRI checklist for completion. Supplemental File 3 – Staff Questions. Supplementary File 4 – Patient Satisfaction Questions. Supplemental File 4: Inner Setting Measures from the CFIR Framework – Fernandez et al (1-low to 5-high, strongly disagree to strongly agree): (n=8). Supplemental File 5 – Adapted General Organizational Index. [file 43058_2020_75_MOESM1_ESM.docx]

**Supplemental File 1: Components of the Healthy Lifestyle Program at the Dartmouth Weight and Wellness Center**

| **Staff** | **Week** | **Content** | **Staff** | **Week** | **Content** |
| --- | --- | --- | --- | --- | --- |
| Health Coach | Week 1^*^ | Mindfulness, Goal Setting | Registered Dietitian | Week 9 | Detoxing your diet and food tracking |
|  | Week 2 | Hunger Awareness, Mindful Eating |  | Week 10 | Food label reading and serving size |
|  | Week 3 | Working with Emotions |  | Week 11 | Meal planning, grocery shopping, preparing for success |
| Exercise Specialist | Week 4 | Movement vs. Exercise | Exercise Specialist | Week 12 | Sorting through the noise |
| Health Coach | Week 5 | Managing Thoughts | Registered Dietitian | Week 13 | The power of protein |
|  | Week 6 | Stress + Social Support |  | Week 14 | Healthy carbohydrates |
|  | Week 7 | Problem Solving |  | Week 15 | Good/bad fats, review of the toolbox |
| Exercise Specialist | Week 8 | Myths and Truths | Exercise Specialist | Week 16 | Moving forward |

*week 1 occurs after the initial visit at the center

**Supplemental File #2**

**
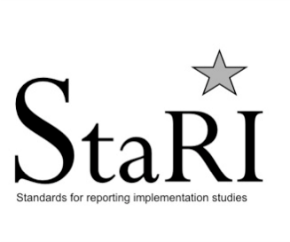
Standards for Reporting Implementation Studies: the StaRI checklist for completion**

The StaRI standard should be referenced as: Pinnock H, Barwick M, Carpenter C, Eldridge S, Grandes G, Griffiths CJ, Rycroft-Malone J, Meissner P, Murray E, Patel A, Sheikh A, Taylor SJC for the StaRI Group. Standards for Reporting Implementation Studies [(StaRI) statement](http://www.bmj.com/content/356/bmj.i6795.full). *BMJ* 2017;356:i6795

The detailed Explanation and Elaboration document, which provides the rationale and exemplar text for all these items is: Pinnock H, Barwick M, Carpenter C, Eldridge S, Grandes G, Griffiths C, Rycroft-Malone J, Meissner P, Murray E, Patel A, Sheikh A, Taylor S, for the StaRI group. Standards for Reporting Implementation Studies [(StaRI). Explanation and Elaboration document](http://bmjopen.bmj.com/content/7/4/e013318.full?ijkey=vv4LKZxc25YcLJv&keytype=ref). *BMJ Open* 2017 2017;7:e013318

Notes: A key concept of the StaRI standards is the dual strands of describing, on the one hand, the implementation strategy and, on the other, the clinical, healthcare, or public health intervention that is being implemented. These strands are represented as two columns in the checklist.

| The primary focus of implementation science is the implementation strategy (column 1) and the expectation is that this will always be completed. | The evidence about the impact of the intervention on the targeted population should always be considered (column 2) and either health outcomes reported or robust evidence cited to support a known beneficial effect of the intervention on the health of individuals or populations. |
| --- | --- |

The StaRI standardsrefers to the broad range of study designs employed in implementation science. Authors should refer to other reporting standards for advice on reporting specific methodological features. Conversely, whilst all items are worthy of consideration, not all items will be applicable to, or feasible within every study.

| **Checklist item** | | **Reported on page #** | **Implementation Strategy** | **Reported on page #** | **Intervention** |
| --- | --- | --- | --- | --- | --- |
|  | |  | “Implementation strategy” refers to how the intervention was implemented |  | “Intervention” refers to the healthcare or public health intervention that is being implemented. |
| **Title and abstract** | | | | | |
| Title | **1** | 1 | Identification as an implementation study, and description of the methodology in the title and/or keywords | | |
| Abstract | **2** | 2 | Identification as an implementation study, including a description of the implementation strategy to be tested, the evidence-based intervention being implemented, and defining the key implementation and health outcomes. | | |
| **Introduction** | | | | | |
| Introduction | **3** | 4 | Description of the problem, challenge or deficiency in healthcare or public health that the intervention being implemented aims to address. | | |
| Rationale | **4** | 4 | The scientific background and rationale for the implementation strategy (including any underpinning theory/framework/model, how it is expected to achieve its effects and any pilot work). | 4 | The scientific background and rationale for the intervention being implemented (including evidence about its effectiveness and how it is expected to achieve its effects). |
| Aims and objectives | **5** | 4 | The aims of the study, differentiating between implementation objectives and any intervention objectives. | | |
| **Methods: description** | | | | | |
| Design | **6** | 5 | The design and key features of the evaluation, (cross referencing to any appropriate methodology reporting standards) and any changes to study protocol, with reasons | | |
| Context | **7** | 5 | The context in which the intervention was implemented. (Consider social, economic, policy, healthcare, organisational barriers and facilitators that might influence implementation elsewhere). | | |
| Targeted ‘sites’ | **8** | 5 | The characteristics of the targeted ‘site(s)’ (e.g locations/personnel/resources etc.) for implementation and any eligibility criteria. |  | The population targeted by the intervention and any eligibility criteria. |
| Description | **9** | 6 | A description of the implementation strategy | 6 | A description of the intervention |
| Sub-groups | **10** |  | Any sub-groups recruited for additional research tasks, and/or nested studies are described | | |
| **Methods: evaluation** | | | | | |
| Outcomes | **11** | 7 | Defined pre-specified primary and other outcome(s) of the implementation strategy, and how they were assessed. Document any pre-determined targets | 7 | Defined pre-specified primary and other outcome(s) of the intervention (if assessed), and how they were assessed. Document any pre-determined targets |
| Process evaluation | **12** | 7-8 | Process evaluation objectives and outcomes related to the mechanism by which the strategy is expected to work | | |
| Economic evaluation | **13** | n/a | Methods for resource use, costs, economic outcomes and analysis for the implementation strategy | n/a | Methods for resource use, costs, economic outcomes and analysis for the intervention |
| Sample size | **14** | n/a | Rationale for sample sizes (including sample size calculations, budgetary constraints, practical considerations, data saturation, as appropriate) | | |
| Analysis | **15** | 8 | Methods of analysis (with reasons for that choice) | | |
| Sub-group analyses | **16** | n/a | Any a priori sub-group analyses (e.g. between different sites in a multicentre study, different clinical or demographic populations), and sub-groups recruited to specific nested research tasks | | |

| **Results** | | | | | |
| --- | --- | --- | --- | --- | --- |
| Characteristics | **17** | 8-9 | Proportion recruited and characteristics of the recipient population for the implementation strategy | 8-9 | Proportion recruited and characteristics (if appropriate) of the recipient population for the intervention |
| Outcomes | **18** | 8-9 | Primary and other outcome(s) of the implementation strategy | 8-9 | Primary and other outcome(s) of the Intervention (if assessed) |
| Process outcomes | **19** |  | Process data related to the implementation strategy mapped to the mechanism by which the strategy is expected to work | | |
| Economic evaluation | **20** | n/a | Resource use, costs, economic outcomes and analysis for the implementation strategy | n/a | Resource use, costs, economic outcomes and analysis for the intervention |
| Sub-group analyses | **21** | n/a | Representativeness and outcomes of subgroups including those recruited to specific research tasks | | |
| Fidelity/ adaptation | **22** | na | Fidelity to implementation strategy as planned and adaptation to suit context and preferences | n/a | Fidelity to delivering the core components of intervention (where measured) |
| Contextual changes | **23** | 9-10 | Contextual changes (if any) which may have affected outcomes | | |
| Harms | **24** | n/a | All important harms or unintended effects in each group | | |
| **Discussion** | | | | | |
| Structured discussion | **25** | 10-14 | Summary of findings, strengths and limitations, comparisons with other studies, conclusions and implications | | |
| Implications | **26** | 10-14 | Discussion of policy, practice and/or research implications of the implementation strategy (specifically including scalability) | 10-14 | Discussion of policy, practice and/or research implications of the intervention (specifically including sustainability) |
| **General** | | | | | |
| Statements | **27** | 1 | Include statement(s) on regulatory approvals (including, as appropriate, ethical approval, confidential use of routine data, governance approval), trial/study registration (availability of protocol), funding and conflicts of interest | | |

**Supplemental File #3 – Staff Questions**

**Questions:**

Assess the intervention’s impact on the processes of care and important stakeholders (Section Chief, team leaders, administrator, practice manager, administrative supervisor) and clinical staff (medical coders, nursing, secretarial, desk, and others) involved in patient care.

1. ‘buy-in’ and/or promotion of the intervention

- What did you think of the current study?
- How did you think this could be helpful for participants?
  - If not, why not?
- Were you in favor of the current study?
  - If so, why?
  - If not, why not?

1. support and personal satisfaction of intervention delivery

- did you feel that things went well in its delivery/
  - if so, why?
  - If not, why not?
- Did you feel participants were satisfied with the intervention
  - if so, why?
  - If not, why not?

1. potential to improve care quality

- how do you see this study improving care in the future?
- Is there potential for improvement in the quality of care?

1. interruptions in patient/provider workflow

- did this study lead to problems in the usual clinical workflow or operations?
  - if so, why?
  - If not, why not?

1. an effect on work processes:

- can you describe how this study impacted the following:
  - workflow (# patients treated)
  - ii. staff (change in work distribution/task shifting);
  - iii. training (time spent on training to perform work);
  - iv. resources (change in work hours);

1. an impact on cultural outcomes (staff attitudes and experiences) towards the pilot

- can you describe the attitudes of other staff members towards this pilot
- can you describe the experiences of the other staff members towards this pilot

1. an impact on the organizational aspects of Telemedicine

- can you describe how this pilot could be sustainable in the future
- do you think telemedicine is usable in this population
  - how about from a provider’s standpoint?
- Does telemedicine increase the amount of time needed to care for patients
- Is the clinic ready to adopt this delivery system?
  - if so, why?
  - If not, why not?

h. do you think there is value in telemedicine

Do you have any final thoughts about how we could improve the program?

**Supplementary File #4 – Patient Satisfaction Questions**

As I mentioned, we are trying to figure out how we can best meet the needs of people like youf and how we can implement this program successfully in the future.

- Why did you decide to enroll in this study?
- When you were told about the program, what were you hoping to get out of it?

The first session was meant to explain the program, how it works, and provide general overviews and examples/goals for next steps. This was completed by our Research Staff

- How clear was that first session- do you feel like you understood how the program could help you?
- Was this explained in a way that made sense to you or did it sound like a lot of jargon/unfamiliar language?
- If you had to explain to a friend what the program is and how it works, what would you tell him or her?

Overall, what did you think of this video-conferencing-based study?

- Can you tell me how you have felt about doing the program?
- Can you give me particular examples of something you liked?
- Can you give me particular examples of something that you disliked?
- How was the timing of this- would you have found this program helpful earlier on or was it best to start now?
- Are there things that we could add to the program that would make it better?
- What did you learn about yourself from being in this program?
- Can you describe whether this study was acceptable to participants
  - If so, describe why?
  - If not, describe why?
- Did you attend all the sessions?
  - If so, why was this so?
  - If not, what prevented you from doing so?

Now I’d like to ask you some specific questions about the parts of the program.

- Were the number of video-based visits just right, too many or too few
- Other studies have done more or fewer sessions, and/or met as a group first, then individually. Would that be of interest to you?
  - Elaboration needed here
- Did it feel flexible- focused on what was important to you and meeting your needs?
  - *If not:* what would have helped- what would have met your needs better?
- Can you describe whether the tools used in the health coaching were helpful?
- How do you think you will continue to use the tools that you learned through the program?
- What do you think were the effects of this program on your health?
- Are there missing features in this study that you would like to see?
- How do you think the intervention impacted one’s health status

**Videoconferencing**

- What do you think some of the advantages were of participating in a video-based program
  - Why?
- What are some of the disadvantages of participating in a video-conferencing based program.
  - Why?
- Were there difficulties with using the video-conferencing? If so, can you describe what?
- Do you have any advice for improvement?
- Can you describe whether there were any privacy concerns
- How do you think video-conferencing can assist rural adults?

**Supplemental File 4:** Inner Setting Measures from the CFIR Framework – Fernandez et al ( 1-low to 5-high, strongly disagree to strongly agree): (n=8)

| **Organizational Culture** | Mean +/- SD | Median | Range |
| --- | --- | --- | --- |
| People at all levels openly talk about what is and isn’t working | 4.3±1.0 | 4.5 | 2-5 |
| Most people in this clinic are willing to change how they do things in response to feedback from others | 4.3±0.7 | 4.0 | 3-5 |
| It is hard to get things to change in our clinic | 3.4±0.9 | 4.0 | 2-4 |
| I can rely on the other people in this clinic to do their jobs well | 4.9±0.4 | 5.0 | 4-5 |
| Most of the people who work in our clinic seem to enjoy their work | 4.6±0.5 | 5.0 | 4-5 |
| Difficult problems are solved through face-to-face discussions | 4.5±0.5 | 4.5 | 4-5 |
| We regularly take time to reflect on how we do things | 4.6±0.5 | 5.0 | 4-5 |
| After trying something new, we take time to think about how it worked | 4.5±0.5 | 4.5 | 4-5 |
| People in this clinic operate as a real team | 4.9±0.4 | 5.0 | 4-5 |

| **Culture Stress** | Mean +/- SD | Median | Range |
| --- | --- | --- | --- |
| I am under too many pressures to do my job effectively | 2.8±0.9 | 2.5 | 2-4 |
| Staff members often show signs of stress and strain | 3.5±0.8 | 4.0 | 2-4 |
| The heavy workload here reduces program effectiveness | 3.0±0.9 | 3.0 | 2-4 |
| Staff frustration is common here | 3.0±1.1 | 3.0 | 1-4 |

| **Culture Effort** | Mean +/- SD | Median | Range |
| --- | --- | --- | --- |
| People in this clinic always want to perform to the best of their abilities | 4.8±0.5 | 5.0 | 4-5 |
| People are enthusiastic about their work | 4.8±0.5 | 5.0 | 4-5 |
| People in our clinic get by with doing as little as possible | 4.8±0.5 | 5.0 | 4-5 |
| People are prepared to make a special effort to do a good job | 4.6±0.5 | 5.0 | 4-5 |
| People in this clinic do not put more effort into their work than they have to | 4.6±0.5 | 5.0 | 4-5 |

| **Implementation Climate** | Mean +/- SD | Median | Range |
| --- | --- | --- | --- |
| Clinic staff are expected to help the SYNERGY Telehealth Project meet its goal | 4.2±0.5 | 4.0 | 4-5 |
| Clinic staff gets the support they need to implement the SYNERGY Telehealth Project | 3.5±0.9 | 3.5 | 2-5 |
| Clinic staff gets recognition for implementing the SYNERGY Telehealth Project | 3.5±0.5 | 3.5 | 3-4 |
| The SYNERGY Telehealth Project is a top priority of the clinic | 2.9±1.0 | 2.5 | 2-4 |
| **Learning Climate** | Mean +/- SD | Median | Range |
| We regularly take time to consider ways to improve how we do things | 4.0±1.1 | 4.0 | 2-5 |
| People in our clinic actively seek new ways to improve how we do things | 4.4±0.5 | 4.0 | 4-5 |
| This clinic encourages everyone to share ideas | 4.5±0.5 | 4.5 | 4-5 |
| This clinic learns from its mistakes | 4.1±0.8 | 4.0 | 3-5 |
| When we experience a problem in the clinic, we make a serious effort to figure out what’s really going on | 4.3±0.7 | 4.0 | 3-5 |
| **Leadership Engagement** | Mean +/- SD | Median | Range |
| The clinic leadership makes sure that we have the time and space necessary to discuss changes to improve care | 3.0±1.2 | 3.5 | 1-4 |
| Leadership in this clinic creates an environment where things can be accomplished | 3.6±0.5 | 4.0 | 3-4 |
| Clinic leadership promotes an environment that is an enjoyable place to work | 3.5±1.1 | 4.0 | 2-5 |
| Leadership strongly supports clinic change efforts | 3.4±0.7 | 3.5 | 2-4 |

| **Available Resources** | Mean +/- SD | Median | Range |
| --- | --- | --- | --- |
| In general, when there is agreement that change needs to happen in the clinic, we have the necessary support in terms of budget or financial resources | 2.6±0.7 | 2.5 | 2-4 |
| In general, when there is agreement that change needs to happen in the clinic, we have the necessary support in terms of training | 3.1±0.8 | 3.0 | 2-4 |
| In general, when there is agreement that change needs to happen in the clinic, we have the necessary support in terms of staffing | 2.1±1.0 | 2.0 | 1-4 |
| The following are available to make the SYNERGY Telehealth Project work in our clinic – equipment and materials | 3.4±0.9 | 4.0 | 2-4 |
| The following are available to make the SYNERGY Telehealth Project work in our clinic – patient awareness/need | 3.4±0.7 | 3.5 | 2-4 |
| The following are available to make the SYNERGY Telehealth Project work in our clinic – provider buy-in | 3.8±0.7 | 4.0 | 3-5 |
| The following are available to make the SYNERGY Telehealth Project work in our clinic – intervention team | 3.8±0.7 | 4.0 | 2-4 |

**Supplemental File 5 – Adapted General Organizational Index**

General Organizational Index (n=8)

- G1: Program Philosophy
  - The program leader (PI) is committed to a clearly articulated philosophy consistent with the specific evidence-based model (yes/no)
  - The Senior Staff (Section Chief/Practice Manager) is committed to a clearly articulated philosophy consistent with the specific evidence-based model (yes/no)
  - The Practitioners (APRN/MDs) providing the evidence-based practice are committed to a clearly articulated philosophy consistent with the specific evidence-based model (yes/no)
  - The Patients receiving the evidence-based practice show a clear understanding of the program philosophy (yes/no)
  - The Written Materials in this study demonstrate a clearly articulated philosophy consistent with the specific evidence-based model. (yes/no)
- G2: Eligibility/Client Identification
  - Standardized screening of who would be an ideal candidate (ie a readiness to change scale)
- G3: Penetration
  - # clients receiving Intervention
  - # clients eligible for Intervention
- G4: Assessment – at each visit assessment
  - # total patients enrolled at each timepoint (baseline, 4, 8, 12, 16)
  - # of survey assessments completed
  - # of objective assessments completed at each time point
- G5: Individualized Treatment Plan
  - Is there EMR documentation for each participant at each visit?
- G6: Individualized treatment – all clients receive individualized treatment plans meeting the goals
- G7: Training – Did all staff receive training
- G8: Supervision
- G9: Process Monitoring
- G10: Outcome Monitoring
  - Were data presented
- G11: Quality Assurance
- G12: Client Choice Regarding Service
